# LIPSHOK: LIARA Portable Smart Home Kit

**DOI:** 10.3390/s22082829

**Published:** 2022-04-07

**Authors:** Kévin Chapron, Florentin Thullier, Patrick Lapointe, Julien Maître, Kévin Bouchard, Sébastien Gaboury

**Affiliations:** Laboratoire d’Intelligence Ambiante pour la Reconnaissance d’Activités, Département d’Informatique et de Mathématiques, Université du Québec à Chicoutimi, 555 Bd. de l’Université, Saguenay, QC G7H 2B1, Canada; kevin.chapron1@uqac.ca (K.C.); patrick.lapointe@hotmail.ca (P.L.); julien1_maitre@uqac.ca (J.M.); kevin_bouchard@uqac.ca (K.B.)

**Keywords:** ambient intelligence, smart home in a box, architecture, framework

## Abstract

Several smart home architecture implementations have been proposed in the last decade. These architectures are mostly deployed in laboratories or inside real habitations built for research purposes to enable the use of ambient intelligence using a wide variety of sensors, actuators and machine learning algorithms. However, the major issues for most related smart home architectures are their price, proprietary hardware requirements and the need for highly specialized personnel to deploy such systems. To tackle these challenges, lighter forms of smart home architectures known as smart homes in a box (SHiB) have been proposed. While SHiB remain an encouraging first step towards lightweight yet affordable solutions, they still suffer from few drawbacks. Indeed, some of these kits lack hardware support for some technologies, and others do not include enough sensors and actuators to cover most smart homes’ requirements. Thus, this paper introduces the LIARA Portable Smart Home Kit (LIPSHOK). It has been designed to provide an affordable SHiB solution that anyone is able to install in an existing home. Moreover, LIPSHOK is a generic kit that includes a total of four specialized sensor modules that were introduced independently, as our laboratory has been working on their development over the last few years. This paper first provides a summary of each of these modules and their respective benefits within a smart home context. Then, it mainly focus on the introduction of the LIPSHOK architecture that provides a framework to unify the use of the proposed sensors thanks to a common modular infrastructure capable of managing heterogeneous technologies. Finally, we compare our work to the existing SHiB kit solutions and outline that it offers a more affordable, extensible and scalable solution whose resources are distributed under an open-source license.

## 1. Introduction

Over the years, various implementations of smart homes have been developed in laboratories or real habitations built for research purposes [1,2,3,4,5,6,7,8]. These works mainly focus on using sensors, effectors and learning algorithms to enable the use of ambient intelligence (Am.I.) as an empirical method in order to support older people’s autonomy and health monitoring for medical purposes. For instance, enhanced homes with Am.I. allow one to improve the safety of residents who suffer from cognitive impairments. At the same time, they help healthcare professionals track a resident’s condition so they are able to adapt their decisions. While each study in the field of smart homes has been developed to address these concerns and more specifically, the activity recognition problem [9], they all suggest different methods and infrastructures to achieve these objectives. Regardless, the major issues for most related smart home architectures are their expensive price, their lack of scalability due to proprietary hardware requirements that are rarely compatible with wearable devices [10] and the highly specialized personnel needed to deploy such systems [6]. The high costs involved in a number of traditional smart home implementations are primarily related to the prices of the required networking hardware and servers rather than the prices of the sensors and actuators themselves, since they remain relatively inexpensive.

In order to tackle these challenges, lighter implementations of smart home architectures known as smart home in a box (SHiB) have been proposed [5,11]. These kits have been designed mainly to offer cheap and easy to install solutions that do not compromise the capabilities of traditional smart home infrastructures. Moreover, these kits do not require any formal training to be operated. While existing SHiB kits remain an encouraging first step towards lightweight yet affordable smart home infrastructures, they still suffer from a few drawbacks. Indeed, these solutions do not always enable the use of various types of sensors or actuators, such as static and wearable devices with high or low sampling rates. Moreover, most of the kits that have been adopted so far are essentially built from a single technology (e.g., a ZigBee mesh network), thereby limiting the support for several other technologies that may also be involved in smart homes.

This paper introduces the LIARA Portable Smart Home Kit (LIPSHOK), a new SHiB kit that is intended to be a better alternative to existing smart home in a box implementations. The main advantage of LIPSHOK is that it is able to manage heterogeneous sensors and actuators, and several wireless communication technologies through a unified modular architecture. Moreover, all the modules that are part of the kit have been designed with the aim of providing a low-cost, self-contained and easy-to-install solution. In addition, since these components are open-source, it is possible for our SHiB kit to be extended with more features than the distributed ones to best suit specific needs, a task that may be cumbersome to achieve with proprietary hardware.

As of now, LIPSHOK comes with four specialized sensors that have been developed by our laboratory to fulfill modern smart home needs and related research. All the details as regards the four hardware components, including bathroom modules [12], a gait speed module [13], PIR-BLE-RSSI modules [14] and a smart wristband module [15], are provided in the next sections.

The rest of the paper is structured as follows: Section 2 presents the current state of smart home in a box kits and provides more insightful details about the motivation for the development of LIPSHOK. Section 3 briefly reports the sensors included in the kit. Next, Section 4 describes the design of the LIPSHOK architecture while Section 5 discusses the benefits of using LIPSHOK. Finally, Section 6 draws conclusions and Section 7 mentions future work we will accomplish.

## 2. Current State of Smart Home in a Box Kits

To the best of our knowledge, very few works have presented self-sufficient, affordable and easy to install smart home in a box kits.

The most popular one is CASAS: the smart home in a box [5]. This kit was developed at Washington State University with the main objectives of being easy to install and affordable. The physical layer offered by this kit is a mesh network composed of both sensors and actuators that communicate with each other through the ZigBee protocol. Each device can be powered by simple batteries while providing long-term functioning. Moreover, new devices may be added to the mesh network on demand, allowing the entire infrastructure to scale seamlessly and automatically. Indeed, the physical layer is linked to a ZigBee bridge that allows each device to communicate with a publish/subscribe manager that composes the messaging service middleware. Finally, an application layer is hosted on a small server where additional computing is performed for data storage and activity recognition.

The implementation proposed by the CASAS kit yields several advantages. First, the use of low-power communication through battery-powered sensors and actuators considerably reduces the cost and simplifies the cumbersome installation process required to make homes smart. Moreover, by its design the CASAS kit remains efficient, simple, scalable and particularly well suited for sensors with low data rates, including binary sensors and actuators (i.e., on/off values), such as a PIR motion sensor. However, the main limitations of this infrastructure is its inability to handle both non-ZigBee-enabled sensors—this technology is mandatory in CASAS—and sensors with high data throughput. Examples are inertial measurement units (IMUs) and positioning sensors based on received signal strength indication (RSSI), as they rely mainly on Wi-Fi or Bluetooth communication technologies.

In addition, ref. [16] have introduced the SPHERE smart home architecture. This smart home kit was designed to enable the use of environmental sensors, wearable devices and real-time video analysis. However, since it has been developed for long-term usage, its installation within existing homes remains challenging and requires qualified personnel. In addition, although special attention has been placed by the authors on the use of affordable consumer hardware, its specifications concerning the use of relatively powerful gateways make it a more expensive architecture than CASAS, although it also offers more possibilities. In that sense, as the SPHERE infrastructure may be seen as a more traditional smart home setup, it will not be discussed here in further detail.

A different team in the same research group was working, at the same time, on the development of an independent SHiB solution: SPHERE in a Box [11]. As a subset of the more comprehensive SPHERE smart home infrastructure, the SPHERE in a Box kit was designed, based on the same two main objectives of the CASAS kit: easy installation and affordability. The SPHERE in a Box kit is more oriented towards wearable devices than CASAS, as specific emphasis was placed on the integration of such devices inside smart homes throughout its presentation paper. Thus, it is capable of working with high-data-rate devices. In this case, a Bluetooth Low Energy (BLE) wristband is used with an embedded IMU. This device then communicates with several gateways strategically placed in the environment to locate the wearer through RSSI and to collect IMU data at a frequency of 25 Hz. Finally, each gateway transmits compressed and encrypted data from one or more wristband once a day to a backup database server through a router that provides a Wi-Fi access point and a 3G/4G cellular link to the Internet.

The main drawback of that solution is the lack of diversity in sensors, due to the limitation of including only wristbands with the kit. That leads us to state that it cannot be used as a fully autonomous smart home kit in its current state. Nevertheless, if the kit were to also contain other high-data-throughput sensors and a few low-data-rate sensors, the gateways would have to be improved first. According to our point of view, other wireless communication technologies than BLE and Wi-Fi would have to be added to the gateways, such as ZigBee or Z-Wave. This would allow the use of less power-consuming sensors and actuators, but it would also make the infrastructure more generic. However, given the authors’ proposed hardware system, such improvements in the SPHERE in a Box kit appear to be perfectly achievable without considerably increasing its costs.

Through the detailed analysis of these two main previously proposed smart home in a box kits, the elements of which are provided by Table 1, the first requirement we identified is the need for such systems to be capable of handling both low and high-data-rate sensors and actuators, since both of them coexist in most current smart home designs [10]. Furthermore, since devices included in these kits may rely on heterogeneous technologies often mandated by the specific data acquisition needs that are used by machine learning applications, it remains important that the proposed solution does not limit itself to the support of a single technology. For example, the CASAS solution requires the use of ZigBee-enabled devices only. In this regard, both commercial and industrial sensors also represent one of the main concerns when designing a SHiB kit. Indeed, commercial sensors, although relatively cheap, rarely offer full control over the data they provide: sometimes the sampling frequency is not adjustable; otherwise, it is impossible to have access to raw data in favor of already pre-processed data. On the other hand, industrial sensors offer, most of the time, absolute control on the output data. However, their prices are not compatible with the constraint of proposing a low-cost smart home in a box kit.

This paper presents LIPSHOK: a framework for a generic smart home in a box kit. The kit includes a total of four custom-made sensors that have been designed by our research laboratory to best address the modern challenges of today’s smart homes requirements [17,18]. The main benefit of LIPSHOK is that it respects all the essential constraints for a SHiB kit which we stated previously. Indeed, it has been designed to be easily deployable in various existing environments without depending on a single technology, and the included devices are all heterogeneous. Some of them are static sensors and others are wearable devices, each of them having its specific hardware and software design, so the kit provides support for a wide variety of technologies. In addition, since the whole kit is distributed under an open-source license, the features are easily customizable and extendable. While each of these four sensors has been presented independently, this paper aims to describe their integration with the LIPSHOK framework in the same controlled environment within our laboratory. All details and materials required to reproduce such a deployment are provided.

## 3. Custom Sensors in LIPSHOK

As LIPSHOK is built on integrating several sensors that have been designed by our laboratory and introduced individually, we consider it important to first provide a brief overview of each one before presenting the framework in more detail.

### 3.1. Bathroom Modules

**Figure 1:**
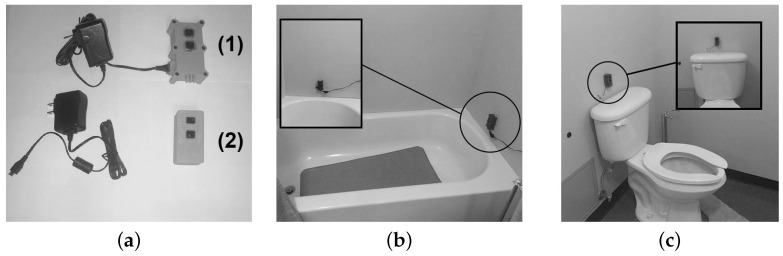
The bathroom modules previously introduced by ref. [12]. (**a**) The bathroom modules where (**1**) refers to the bathtub device and (**2**) is the device for the toilets. (**b**) The first IRPS bathroom device placed in the bathtub. (**c**) The second IRPS bathroom device positioned over the toilets. Reprinted with permission from ref. [12] 2020 IEEE.

Corporal hygiene is a particularly useful indicator for detecting the impairment of the cognitive function of an individual living in a smart home. Indeed, it is known that people affected by a cognitive disorder will spend less and less time taking care of their own hygiene [19,20]. Moreover, in the context of personal hygiene, persons affected by a physical disability take longer to complete activities of daily living (ADLs), such as showering and going to the toilet [21,22], supporting that corporal hygiene remains a reliable indicator of physical condition for residents of smart homes. Hence, the first previously proposed module included in the LIPSHOK SHiB kit is a combination of two devices to be placed in the bathroom [12]. As shown in Figure 1, the two devices are placed both on the bathtub and over the toilet in the bathroom of the smart home.

The bathtub device was roughly waterproofed to ensure minimal safety during the experiments, but it was not made to comply with both dust and water protection standards defined by ingress protection (IP), such as IP67 as defined by the EN-60529 standard (https://keystonecompliance.com/en-60529/ accessed on 22 February 2022). The two modules are based on the same sensor, an infrared proximity sensor (IRPS) (https://www.sparkfun.com/products/8958 accessed on 22 February 2022), allowing each module to compute the distance between the device and a potential human standing in front. This sensor provided accurate presence detection that could not have been achieved with any other sensors that had been reported in the literature. One reason is that passive infrared (PIR) sensors can never be sensitive to someone not moving while standing in front of them [5], and they are not capable of providing the distance. Moreover, the current state-of-the-art reports other types of sensors that are not effective enough at recognizing ADLs adequately, or whether they are being performed by the monitored person or for how long. Among these works, microphone-based recognition [23] and contact-based recognition [24] have only demonstrated the ability to detect the end of an event, providing no further related information.

As detailed in our previous research [12], the reliability of the bathroom modules was evaluated by their ability to accurately identify the activities “taking a shower” and “going to the toilet”, through the use of threshold-based decision algorithms. The data used for this experiment were recorded over a 59-day period by eight participants, all of whom were healthy adults without any motor or cerebral disability. Moreover, a qualitative survey regarding the ease of installation and the acceptability of the bathroom modules by the participants was also conducted. Table 2 exposes an overview of the results obtained for the assessments.

### 3.2. Gait Speed Module

Gait speed is known to be an excellent predictor of diseases such as mild cognitive impairment (MCI) in the older population [25]. More precisely, ref. [26] have demonstrated in a 20-year longitudinal study that although the age has an impact, patients diagnosed with MCI have a further decline in gait speed when compared to the healthy population. However, a possible remediation to help slow and control the progression of such cognitive decline is to consult with occupational therapists for regular monitoring of the gait speed (i.e., each year). Thus, gait speed monitoring appears to be a relevant use case of applied ambient intelligence inside smart homes. In that sense, considering the importance of this predictor which is currently rarely addressed in the literature, our team have been focused on the development of a device to be included in the LIPSHOK SHiB kit as an autonomous module that automatically monitors residents’ gait speed in real time [14].

**Figure 2:**
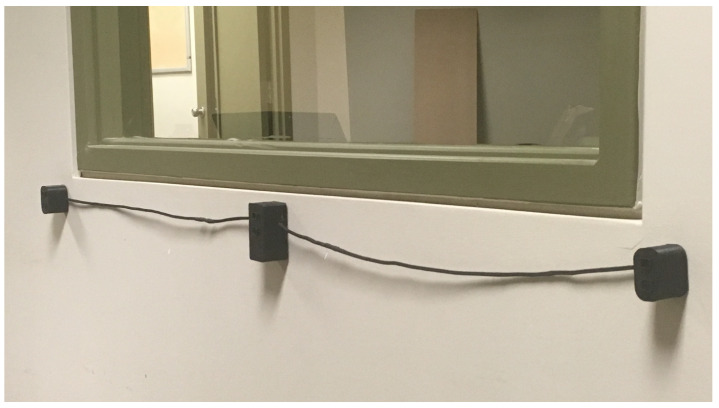
Gait speed module deployed in our smart home laboratory introduced by ref. [14]. Reprinted with permission from ref. [14] 2020 Springer Nature.

The gait speed module was built based on three IRPS sensors, the same as for the bathroom module. Each sensor is placed at a height of 90cm on a hallway wall at a fixed distance of 60cm from the others, resulting in a module with a total length of 120cm, as shown in Figure 2. This gait speed module was designed to be affordable, easy to install and to provide a relatively straightforward way of operating. In a nutshell, whenever a monitored person walks by the module, the detection is monitored in order to allow the computation of the gait speed.

The gait speed module was evaluated in a three-phase experimental procedure which was as close as possible to real use case situations. The procedure was completed by nine participants, all being healthy adults without any known issues in physical condition. The first phase of the experiment focused on the evaluation of the precision of the speed (A) following a 5-meter walk test (5MWT) as introduced by ref. [27]. Then, the second phase of the experiment consisted of the evaluation of the user identification through the BLE RSSI only (B). Finally, the last phase focused on the combination of the identification of monitored persons and activity recognition (C). The data for the activity recognition process were collected with the wristband presented in Section 3.4. Table 3 exposes an overview of the results obtained for such assessments.

### 3.3. PIR-BLE-RSSI Modules

**Figure 3:**
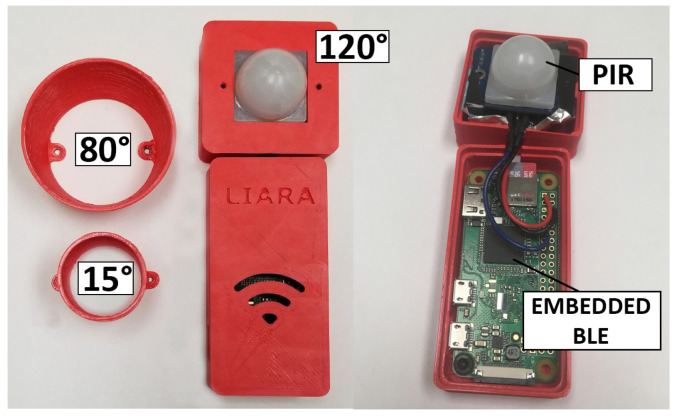
An example of the PIR-BLE-RSSI module introduced by ref. [13]. Reprinted with permission from ref. [13] 2020 Elsevier.

The next module provided in the LIPSHOK SHiB kit is the PIR-BLE-RSSI device (several ones are used). One is shown in Figure 3. This module was designed by our team [13] in order to better address the key challenge when it comes to achieving an accurate activity recognition process in a multi-resident smart home context, namely, effectively associating the sensor observations and the right individuals [28]. Indeed, each PIR-BLE-RSSI module combines a passive-infrared motion sensor and a BLE unit, allowing it to detect movements through more or less restricted fields of view (i.e., 120°, 80° or 15°) and room-level positioning when paired with any wearable device, such as a smartwatch or a smartphone.

The evaluation was performed by installing multiple PIR-BLE-RSSI modules in our smart home laboratory of 43 m${}^{2}$. Next, several realistic scenarios of ADLs were completed by eight participants, all being healthy adults, in a multi-resident setup. Firstly, two monitored persons ($P_{A}$ and $P_{B}$) were equipped with one wearable device each and asked to perform ADLs simultaneously in different areas of the smart home (e.g., $P_{A}$: *washing hands* in the bathroom and $P_{B}$: *changing clothes* in the bedroom). Then, to collect some control data, a third monitored person was requested to stand in the same area without wearing a device for all scenarios in the experiments. Table 4 exposes an overview of the results obtained, outlining the robustness of our system when using multiple PIR-BLE-RSSI modules.

### 3.4. Wristband Module

Over the past few years, wearable devices have been widely used in smart homes to address several research problems in various areas. Indeed, given their small size, their cheap price and their convenience of use and integration in intelligent environments, they have allowed researchers to suggest new systems for continuous health monitoring and the recognition of activities, gestures or falls [29,30,31,32]. In addition, wearable devices have also been shown to provide an excellent basis for addressing the multi-occupancy challenge, as they are capable of both tracking and identifying people, but also recognizing activities in an autonomous manner [33,34,35].

**Figure 4:**
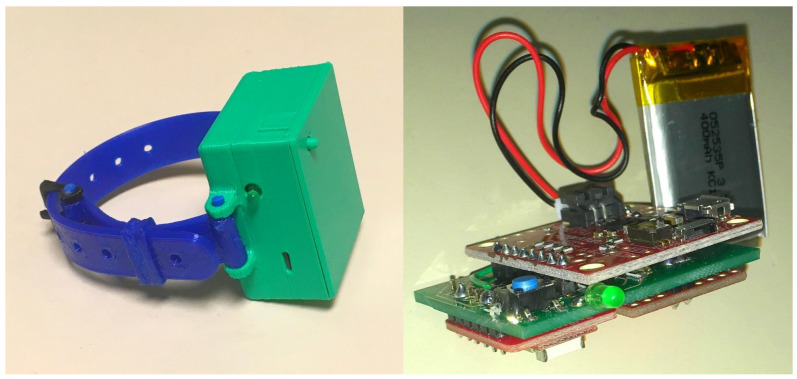
The wristband module introduced by ref. [15]. Reprinted with permission from ref. [15] 2021 IEEE.

Our team focused on developing a wristband module to help with the rehabilitation of people affected by myotonic dystrophy type 1 (DM1) [15,36]—a hereditary neuromuscular disease that causes a variety of impairments, particularly muscle weakness—since it is a prevalent disease in the area of our University (i.e., Saguenay-Lac-Saint-Jean region of Quebec, Canada). As pictured in Figure 4, the proposed wristband hardware relies on a nRF52832 board (https://www.adafruit.com/product/3406 accessed on 22 February 2022) that embeds a native-Bluetooth chip to which a 9-degree-of-freedom (DoF) IMU has been added. However, three of the nine degrees of freedom corresponding to the magnetometer were deactivated, because in the context of activity recognition, it was determined that they did not provide enough relevant information, and therefore may be ignored, especially for devices with limited hardware [37].

In the context of LIPSHOK, the wristband is the most powerful module included in the SHiB kit. Indeed, the wristband module is the only one capable of accurately achieving the recognition of ADLs directly on its embedded hardware through a machine learning algorithm (i.e., a C4.5 decision tree) using the inertial data. However, while every other module presented in this paper is designed to work autonomously, as they are capable of performing simple activity detection on their own, combining their data with the data produced by the wristband can significantly improve the accuracy of the overall system for all the use cases that have been presented. For instance, the gait speed module, the PIR-BLE-RSSI, and the bathroom modules all have the ability to scan wristband identifiers and associate the readings with the nearest person based on an RSSI localization. As regards the recognition of ADLs through inertial data, an overall **F**-measure of 84.40% was obtained during the following experiment: Twenty participants were asked to perform, three times a week, a 5-activity training program (i.e., running, sit-to-stand, stand-to-sit, inactive and walking).  The participants were divided into two equal groups of 10 people according to whether they had to complete the program with the wristband or without, in order to form a control group [15].

## 4. The Design of LIPSHOK

**Figure 5:**
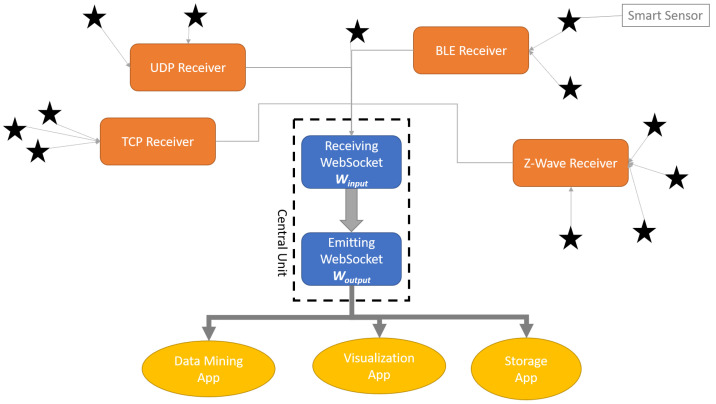
The detailed diagram of the suggested LIPSHOK framework.

In this paper, we introduce LIPSHOK, LIARA Portable Smart Home Kit, a smart home in a box framework. We were motivated by the need to integrate the modules presented in the previous section into a standalone kit that is easy to install and inexpensive. As illustrated in Figure 5, the design of such a framework remains simple. Regarding our implementation, this architecture relies on a centralized unit that does not require powerful hardware to manage the flow of data generated by all the sensors a smart home may involve. Indeed, despite most of the implementations suggested in the literature [8], we have rather opted for the use of a Raspberry Pi 3B, one of the most well-known nanocomputers currently available on the market, since it offers an excellent price–performance ratio.

Moreover, as the proposed architecture is intended for a real-time usage of the data, it therefore avoids the need for expensive storage devices, and it also provides better privacy and security for the residents of smart homes. Nevertheless, the such a framework does not enforce the use real-time data. As it stands, it is possible to create a dedicated application within the LIPSHOK architecture in order to store incoming data in a database server located in another environment.

### 4.1. Main Architecture

The central unit of the LIPSHOK framework hosts the core software component that mainly relies on the use of the websocket protocol (https://www.rfc-editor.org/rfc/inline-errata/rfc6455.html accessed on 22 February 2022). This technology is preferred, since it allows us to create a bidirectional communication channel that is kept open as long as required. In addition, websockets remain simple, well suited for real-time applications and allow one-to-many connections, which is particularly useful when it comes to supporting many smart home sensors.

In the LIPSHOK achitecture framework, there are two distinct websockets, the first one being required to acquire data transmitted by every receiver and the second one being required to emit aggregated data to external clients, such as dedicated applications (e.g., a visualization application) or other devices (e.g., a database server). In order to facilitate future explanations, it is first important to provide the definitions of some key concepts:

- **Smart sensor**: A smart sensor encompasses a physical sensor and a programmable chip, or a nanocomputer, that allows one to embed algorithms or any data processing software, and that provides either a wired or a wireless connectivity (e.g., BLE).
- **Receiver**: A receiver is an entity authorized to collect data of one or more sensors communicating with the same protocol (e.g., Z-Wave). Its only requirement is to be capable of connecting to both a websocket protocol and its related communication protocol. For example, the BLE receiver must be able to connect to any of the BLE smart sensors available and to send their data through the websocket protocol.
- **$W_{\mathrm{input}}$** is the notation employed to refer the receiving websocket of the central unit. Its role is to combine and normalize data transmitted by the receivers. Moreover, it is also possible for this receiver to act as the default receiver for any smart sensor using a websocket as its only communication protocol.
- **$W_{\mathrm{output}}$** is the notation employed to refer the emitting websocket of the central unit. Its role is to stream aggregated and normalized data obtained from smart sensors through $W_{input}$ to every connected client. Therefore, if two interfaces are connected to $W_{output}$, they will both receive real-time data, regardless of the underlying communication protocol required by each sensor.

While our implementation suggests hosting the receivers within the same central unit, it is simple to see how the system may be improved by distributing them across multiple physical devices, as long as they have the proper requirements. The architecture should then benefit from better load distribution across the network and better robustness against single points of failure (SPoF).

### 4.2. Protocol Receivers

In order to provide a generic architecture capable of adapting to most technologies being used by sensors and actuators of smart homes, the suggested design of the LIPSHOK framework defines various software components as receivers. Every receiver operates on a specific network port. Each one is associated with a given technology, as detailed in Table 5. While these receivers cover most of the most popular technologies, including the ones required for every module included in the kit, more receivers may be added to the framework. Indeed, as an open-source project (https://github.com/kevinchapron/LIPSHOK-final accessed on 22 February 2022), the LIPSHOK framework was designed to be as extensible as possible. In addition, while the core software is written in GoLang (https://golang.org/ accessed on 22 February 2022), additional features may be developed in other languages without compromising its proper functioning, as long as they comply with the architectural guidelines related to port forwarding and encryption.

### 4.3. Security

**Figure 6:**
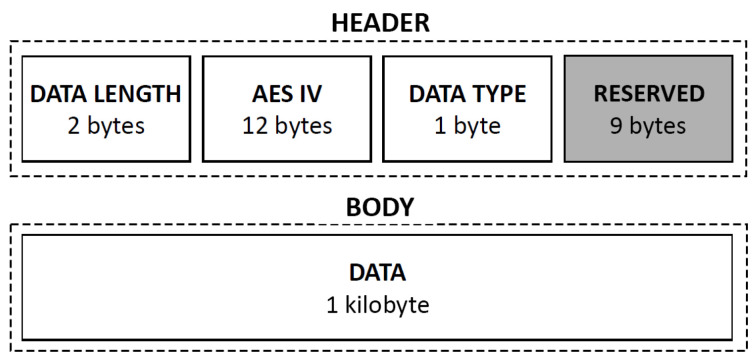
The structure of a message packet in the LIPSHOK architecture.

The main requirement mandated by the LIPSHOK framework is the need for the data transmission process to implement a previously defined encryption layer. This is to prevent malicious users from acquiring sensible data that are easily usable through a man in the middle (MITM) attack [38]. Therefore, the suggested implementation is based on the advanced encryption standard (AES) algorithm. Thus, each message transmitted within the architecture must comply with the packet structure illustrated in Figure 6. Since the AES-128 master key is generated only one time, it is then stored securely in the central unit and in each module included in the kit. Moreover, to reduce the threats of potential packet interception, a unique AES initialization vector (IV) is generated for each new message.

While AES is a powerful yet efficient encryption algorithm, some modules included in the kit, such as BLE-based devices (e.g., the wristband), limit the size reserved for the body of the message to 20 bytes, making them not suitable with AES encryption. To cope with such a problem, we therefore suggest using the PRESENT algorithm [39] since it is a lightweight block cipher encryption method advertised to be 2.5 times more cost-effective than AES (https://nieuws.kuleuven.be/en/content/2012/ultra-lightweight-encryption-method-becomes-international-standard accessed on 22 February 2022). The PRESENT algorithm uses an 8 byte block cipher. In that sense, as data transmitted by BLE-based devices within the architecture are 20 bytes long, the encryption algorithm is applied three times using the following bytes: [0,8], [6,14] and [12,20]. A total of four bytes need to be overlapped to fit the algorithm. However, this overlap has no impact, since the decryption operation is applied in reverse order.

## 5. Why Use Lipshok?

From our point of view, the LIPSHOK SHiB kit detailed in this paper should benefit all stakeholders concerned with the use and development of intelligent environments. Inspired by the early proposal of ref. [5], the kit has also been designed to be easy to install in either new or already existing homes regardless of the available setup, for a very low price. For instance, Table 6 provides an evaluation of the cost for each module included in the LIPSHOK kit and the infrastructure costs. Furthermore, Table 7 offers a costs comparison of LIPSHOK with related SHiB solutions (i.e., CASAS [1] and SPHERE in a Box [11]) when deployed in a one-bedroom apartment. However, it must be noted that quoted prices for the LIPSHOK kit represent the costs for the production of the proofs of concept. Large-scale manufacturing of the different modules is expected to reduce significantly these costs.

When compared to existing SHiB kits, such as CASAS and SPHERE in a Box, LIPSHOK is the most affordable when taking into account the number of sensors it provides out of the box. Furthermore, since the architecture has been made to allow integrating sensors and actuators based on various technologies and thus working at several data rates, the kit features better extensibility than SPHERE in a Box by default. In addition, having the entire LIPSHOK infrastructure (i.e., hardware blueprints and the firmware and algorithms) distributed under an open-source license also ensures enhanced extensibility, as it allows developers and researchers to easily upgrade the core features of the kit to best suit their needs.

**Figure 7:**
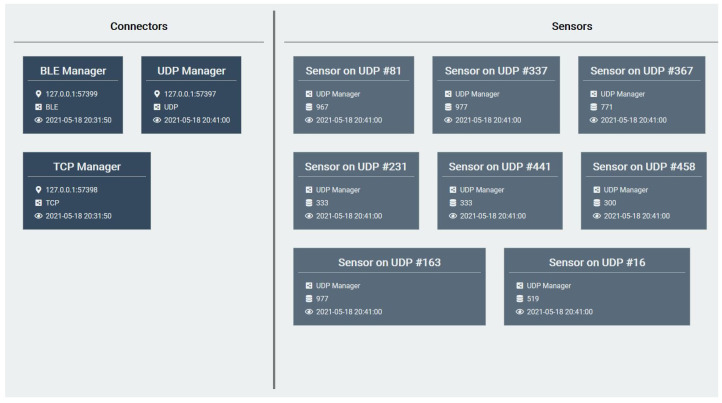
The sensors-state-monitoring application interface included with the LIPSHOK SHib kit.

Finally, LIPSHOK includes everything required from sensors to client applications enabling a fully operational smart home within a couple of hours of installation and configuration. Figure 7 shows the sensors-state-monitoring client application also provided as part of the kit (provisioned on the central unit by default). Additionally, it is important to note that the architecture may also be scaled-up to further meet high-availability requirements in order to improve fault tolerance in the same way as defined by refs. [8,10].

## 6. Conclusions

In this paper, we have introduced the LIARA Portable Smart Home Kit (LIPSHOK), a smart home in a box (SHiB) kit capable of managing both heterogeneous sensors and actuators, along with several wireless communication technologies, in a unified modular architecture. In order to keep this architecture affordable, its design relies on a centralized unit that does not require powerful hardware to manage the flow of data generated by all the sensors in real time. Since it is a simple architecture that is based on inexpensive hardware, we have put our efforts toward offering a standalone and generic solution, in order to facilitate its installation. Moreover, four custom sensors are also featured in this kit. However, these devices do not exclude the possibility for researchers and developers to integrate other sensors or actuators, which may be either proprietary or custom-made in the LIPSHOK kit. While each of these four sensors has been presented independently, this is, to the best of our knowledge, the first time such a comprehensive SHiB kit has been presented.

## 7. Future Work

Future work will focus first on the refection and enhancement of the bathroom modules. Indeed, since these modules have not been designed to meet any of the IP standards, it seems important to offer modules that are IP67 considering the environment where they are installed. Moreover, the need for these modules to be powered through an electric cable, while it only outputs 5V, remains a drawback in their design that may raise concerns for smart home residents. Thus, we are working on making the bathroom modules capable of being powered by a button cell battery.

In addition, as a post-COVID-19 pandemic context is starting to emerge, our objective of deploying the complete LIPSHOK kit in real conditions, within a non-research laboratory environment, now appears achievable. Therefore, future work will focus on this essential step that is required to ensure the LIPSHOK SHiB kit works in residential settings.

## Figures and Tables

**Table 1:** Elements of existing SHiB kits.

	CASAS [1]	SPHERE in a Box [11]
**Main Technology**	ZigBee	BLE
**Included Modules**	IR motion/light sensors (×24) Door sensor (×1) Temperature sensors (×2) Relays (×2)	Wristband (×1)
**Ease of Installation**	Yes no wiring required: battery-powered modules only.	Yes evaluated through a survey conducted by the authors.
**Affordability**	Moderate	High
**Extensibility**	No only compatible with ZigBee enabled modules.	Yes BLE and WiFi compatible out of the box. Possibility to add support for other technologies relatively easily.
**Scalability**	Yes	Yes
**Sensor Data Rates**	only low data rates	both low and high data rates out of the box

**Table 2:** Overview of the results obtained with the bathroom modules [12].

Evaluation	Toilets	Shower/Bathtub
Activity recognition (**F**-measure)	95.26%	98.62%
Duration differences (% of difference)	3.90%	6.48%
Ease of installation (% of agreement)	91.43%	91.43%
Acceptability (% of positive response)	84.38%	90.63%

**Table 3:** Overview of the results obtained with the gait speed module [14].

Evaluation	Result
(A) speed precision (% of precision)	93.38%
(B) raw identification of monitored persons (% of accuracy)	48.00%
(C) identification of monitored persons and activity recognition aggregated (% of accuracy)	84.00%

**Table 4:** Overview of the results obtained for the identification of monitored persons with the PIR-BLE-RSSI module [13].

Identification of Monitored Persons	Result
BLE only	90.22%
BLE and PIR aggregated	92.28%

**Table 5:** Detailed receiver network configurations.

Label	Technology	Port
UDP Receiver	UDP	5010
TCP Receiver	TCP	5020
BLE Receiver	BLE	5030
Z-Wave Receiver	Z-Wave	5040
Main Receiver ($W_{input}$)	Websocket	5001
Main Receiver ($W_{output}$)	Websocket	5003

**Table 6:** Summary of the cost for every module included in the LIPSHOK SHiB kit and for the hardware required to implement the architecture.

Module	Content	Qty.	Unit Price ($US)
**LIPSHOK infrastructure**	Raspberry Pi 3B+ board	1	35.00
	Minimal Raspberry equipment (Minimal equipment for the Raspberry Pi board includes a power cable and a class 10 micro SD memory card with a storage capacity of 32 GB.)	1	30.00
	ZigBee dongle	1	30.00
	Z-Wave dongle	1	60.00
	Total		155.00
**Bathroom modules**	Raspberry Pi Zero W board	2	10.00
	Minimal Raspberry equipment	2	20.00
	16-bit ADC	2	15.00
	IRPS sensor	2	15.00
	Total		120.00
**Gait speed module**	Raspberry Pi Zero W board	1	10.00
	Minimal Raspberry equipment	1	20.00
	16-bit ADC	1	15.00
	IRPS sensor	3	15.00
	Total		90.00
**PIR-RSSI module**	Raspberry Pi Zero W board	1	10.00
	Minimal Raspberry equipment	1	20.00
	PIR sensor	1	10.00
	Total		40.00
**Wristband module**	RedBear BLE Nano V2 board	1	15.00
	LSM9DS1 IMU sensor	1	17.50
	LiPo battery manager	1	21.50
	400 mAh LiPo battery	1	5.50
	Total		59.50

**Table 7:** Cost comparison of two related SHiB kits, CASAS [1] and SPHERE in a Box [11], with LIPSHOK.

SHiB kit	Content	Total Cost ($US)
**CASAS [1]**	Server (×1)	2765.00
	IR motion/light sensors (×24)	
	Door sensor (×1)	
	Relays (×2)	
	Temperature sensors (×2)	
**SPHERE in a Box [11]**	Cellular router (×1)	500.00
	WiFi gateways (×4)	
	Wristband (×1)	
**LIPSHOK**	Central unit (×1)	624.50
	Bathroom modules (×1)	
	Gait speed module (×1)	
	PIR-BLE-RSSI modules (×5)	
	Wristband module (×1)	

## Data Availability

Not applicable.

## Abbreviations

The following abbreviations are used in this manuscript:

5MWT	5-Meter Walk Test
ADL	Activities of Daily Living
AES	Advanced Encryption Standard
Am.I.	Ambient Intelligence
BLE	Bluetooth Low Energy
DM1	Myotonic Dystrophy type 1
DoF	Degrees-of-Freedom
IMU	Inertial Measurement Unit
IP	Ingress Protection
IRPS	Infrared Proximity Sensor
IV	Initialization Vector
LIPSHOK	LIARA Portable Smart Home Kit
MCI	Mild Cognitive Impairments
MITM	Man in the Middle
PIR	Passive Infrared
RSSI	Received Signal Strength Indication
SHiB	Smart Home in a Box
SPoF	Single Points of Failure
TCP	Transmission Control Protocol
UDP	User Datagram Protocol
